# Functional outcome assessment in bipolar disorder: A systematic literature review

**DOI:** 10.1111/bdi.12775

**Published:** 2019-04-14

**Authors:** Maxine Chen, Heather M. Fitzgerald, Jessica J. Madera, Mauricio Tohen

**Affiliations:** ^1^ Medical Affairs Otsuka Pharmaceutical Development & Commercialization, Inc Princeton New Jersey; ^2^ Medical Affairs – Psychiatry Lundbeck LLC Deerfield Illinois; ^3^ Department of Psychiatry & Behavioral Sciences University of New Mexico Albuquerque New Mexico

**Keywords:** bipolar disorder, cognitive function, neuropsychological tests, patient outcome assessment, patient‐reported outcome measures, psychosocial factors, social adjustment

## Abstract

**Objectives:**

Functional impairment is an important driver of disability in patients with bipolar disorder (BD) and can persist even when symptomatic remission has been achieved. The objectives of this systematic literature review were to identify studies that assessed functioning in patients with BD and describe the functional scales used and their implementation.

**Methods:**

A systematic literature review of English‐language articles published between 2000 and 2017 reporting peer‐reviewed, original research related to functional assessment in patients with BD was conducted.

**Results:**

A total of 40 articles met inclusion criteria. Twenty‐four different functional scales were identified, including 13 clinician‐rated scales, 7 self‐reported scales, and 4 indices based on residential and vocational data. The Global Assessment of Functioning (GAF) and the Functional Assessment Short Test (FAST) were the most commonly used global and domain‐specific scales, respectively. All other scales were used in ≤2 studies. Most studies used ≥1 domain‐specific scale. The most common applications of functional scales in these studies were evaluations of the relationships between global or domain‐specific psychosocial functioning and cognitive functioning (eg, executive function, attention, language, learning, memory) or clinical variables (eg, symptoms, duration of illness, number of hospitalizations, number of episodes).

**Conclusions:**

The results of this review show growing interest in the assessment of functioning in patients with BD, with an emphasis on specific domains such as work/educational, social, family, and cognitive functioning and high utilization of the GAF and FAST scales in published literature.

## 1 INTRODUCTION

A meta‐analysis of 25 studies conducted in 15 countries estimated a pooled lifetime prevalence of 1.06% for bipolar I disorder (BP‐I) and 1.57% for bipolar II disorder (BP‐II).^1^, ^2^ However, US epidemiologic studies suggest that approximately 60% of patients with BP‐I receive mental health treatment, a statistic that is similar to what has been reported for other countries.^3^ This suggests that many individuals with bipolar disorder (BD) may experience continued disability, due in part to lack of treatment intervention, which could potentially limit their functioning and productivity and also decrease overall quality of life for them and their families.

The World Health Organization (WHO) has ranked BD as the 12th leading cause of disability worldwide, and the worldwide prevalence of moderate or severe disability for BD is estimated at approximately 22 million individuals.^4^ Poor functioning is considered a key driver of disability in patients with BD.^5^ The WHO International Classification of Functioning, Disability, and Health describes functioning and disability as multidimensional concepts involving the ability to control physical functions; perform activities related to domains such as self‐care and domestic, occupational, social, and civic life; engage in all aspects of life; and manage aspects of the environment that help or hinder these experiences.^6^, ^7^ Patients with BD report a variety problems in work functioning,^8^ with severe work impairment during a considerable portion of their long‐term course of illness,^9^ and high unemployment rates.^10^ They have decreased social engagement,^11^ weaker family relationships,^12^ and an increased likelihood of being separated, divorced, or widowed.^13^ For many patients with BD, these functional impairments persist into symptomatic remission, leading to difficulties in many aspects of their lives.^14^, ^15^ The negative effects of BD extend to caregivers, who report substantial burden and distress involving relationships and day‐to‐day activities.^16^ Thus, for many patients with BD and their families, functional outcome, measured as the ability to fulfill role expectations in all aspects of life and maintain interpersonal relationships, is at times more important than syndromal outcome.^17^

Although most interventional studies in patients with BD have focused on symptoms, recurrences, and mood states as the primary outcome variable, increasingly, studies are also assessing functioning as a key outcome.^17^ In addition, numerous studies have sought to identify determinants of functional outcome in patients with BD. A variety of correlates of poor functional outcome have been identified, including clinical factors such as lack of treatment adherence, comorbid substance abuse or anxiety disorder, and subsyndromal symptoms; demographic variables such as older age, male sex, and low socioeconomic status; and cognitive dysfunction, particularly verbal memory impairment and executive dysfunction.^5^ The growing interest in the relationship between cognitive and psychosocial function in BD is reflected in several recent reviews on this topic^6^, ^17^, ^18^, ^19^ and compels a better understanding of the tools used for functional assessment in this population. The purpose of this systematic literature review was to identify and describe scales that are used to assess functioning in studies of patients with BD and to gain an understanding of the domains of function that clinicians are measuring.

## 2 METHODS

### 2.1 Information sources and eligibility criteria

This systematic literature review was conducted according to the recommendations outlined in the Preferred Reporting Items for Systematic Reviews and Meta‐Analyses (PRISMA) statement.^20^ A search was performed in BIOSIS Previews, Embase, and MEDLINE for English‐language articles published in peer‐reviewed journals between 1 January 2000 and 6 November 2017. The following search string was applied using the abstract and title as search fields: (“functional impairment” OR “psychosocial outcome” OR “psychosocial functioning” OR “psychosocial treatment” OR “psychosocial impairment” OR “occupational function” OR “occupational impairment” OR “occupational” or “functional disability” OR “psychosocial disability” OR “disability or work” OR “cognition” OR “social cognition” OR “stress” OR “functional remediation” OR “cognitive remediation”) AND (“functional recovery” OR “functional outcome” OR “outcome n/1 [patient or recovery]” AND (“bipolar disorder” OR “bipolar I disorder” OR “bipolar II disorder” OR mania OR manic OR “bipolar mania” OR “bipolar depression” OR “bipolar I disorder” OR “manic psychosis” OR “bipolar disorder” OR “affective disorders, psychotic”) AND (scale* OR measure*). Conference publications (ie, posters, summaries, and abstracts), review articles, notes, letters, book chapters, interactive tutorials, or surveys; publications involving animal or in vitro studies; and publications on pediatric populations were excluded.

### 2.2 Article selection process

**Figure 1:**
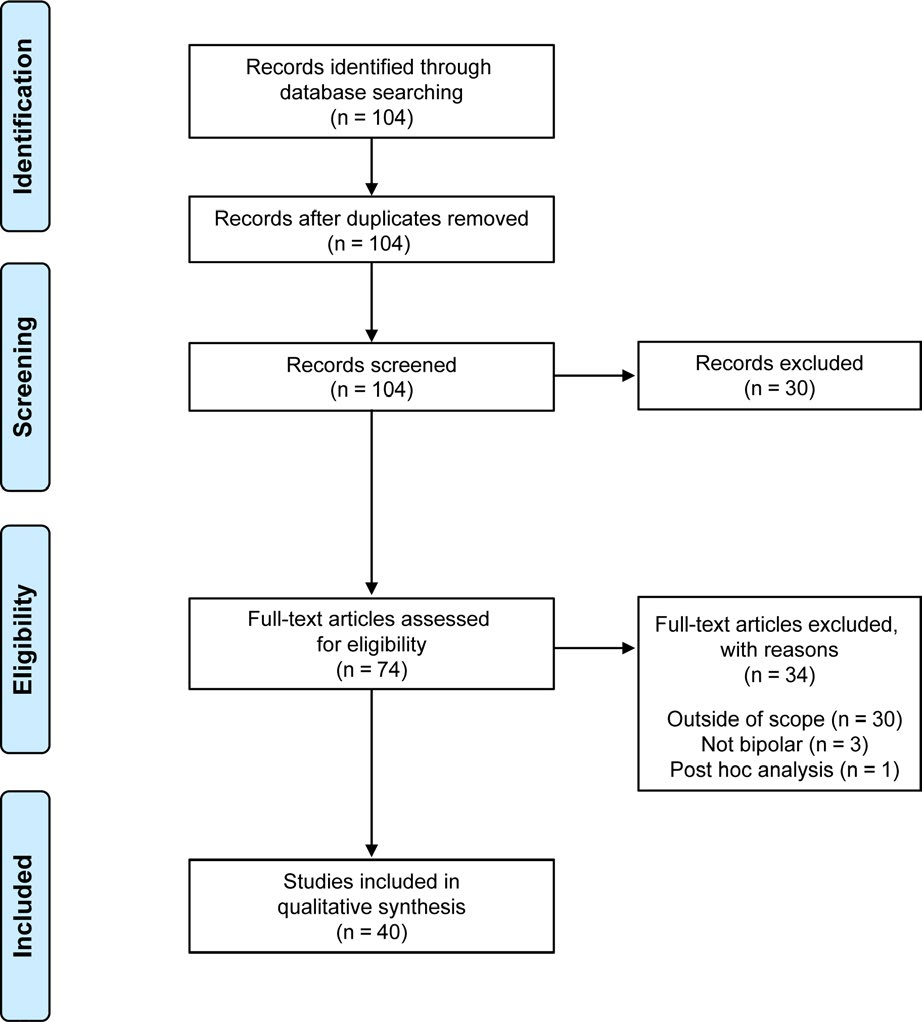
PRISMA flow diagram [Colour figure can be viewed at wileyonlinelibrary.com]

The title and abstract of each retrieved article were independently screened against eligibility criteria by one author. Selected full‐text articles were then divided between the authors for detailed review and inclusion assessment. Figure 1 shows the article selection process.

## 3 RESULTS

### 3.1 Article selection

The search retrieved 104 articles; 30 were determined ineligible based on screening of titles and abstracts, and 74 were selected for full‐text review. Forty articles met the eligibility criteria and were included in the qualitative data analysis (Figure 1).

### 3.2 Risk of publication bias

Several authors and research groups authored multiple articles identified in this review (Table 1 and Table 2), indicating the potential for publication bias.

**Table 1:** Cross‐sectional studies summary

Study	Objective	Patients	Functional SCALE	Functional DOMAINS	Conclusions
GAF					
Konstantakopoulos 2016 Psychiatriki 27:182‐191^27^	To examine the effect of clinical and cognitive factors, such as Theory of Mind, on psychosocial functioning in patients with euthymic BP‐I	‐ 49 patients with euthymic BP‐I ‐ 53 healthy controls matched by sex, age, and educational level	GAF	Psychosocial functioning	Subclinical symptoms and cognitive dysfunction, especially social cognition, negatively affect the psychosocial functioning of patients with BP‐I who are in remission. Theory of Mind mediates the impact of subclinical symptoms and the basic cognitive deficits on social dysfunction seen in patients with euthymic BP‐I
Martinez‐Arán 2002 Psychother Psychosom 71:39‐46^28^	To determine whether neuropsychological variables, especially those related to executive function, have more predictive power for functional outcome than clinical variables, and to ascertain whether patients with BD or schizophrenia show different patterns of impairment with respect to executive function	‐ 49 patients with euthymic BD ‐ 49 patients with schizophrenia	GAF	‐ Psychosocial functioning ‐ Occupational functioning	Patients with schizophrenia showed more cognitive dysfunctions vs remitted patients with BD, but executive functioning was similar in both groups. Verbal fluency may be a useful indicator of global psychosocial functioning in patients with euthymic BD
Martinez‐Arán 2002 Neuropsychobiology 46:16‐21^29^	To ascertain whether neuropsychological performance is similar between patients with BP‐I or BP‐II and depression and patients with euthymic BP‐I or BP‐II, and to determine if neuropsychological variables are related to psychosocial functioning	‐ 30 patients with BP‐I or BP‐II and depression ‐ 30 patients with euthymic BP‐I or BP‐II	GAF	Psychosocial functioning	The two groups had similar scores in most neuropsychological measures, including attention, memory, and executive function, despite differences in clinical symptomatology severity. Verbal fluency correlated with social functioning in patients with euthymic BP‐I and BP‐II. The difficulties observed in patients with BD during remission may relate to cognitive impairment in verbal fluency function, even after controlling for low levels of affective symptoms
Martinez‐Arán 2004 Am J Psychiatry 161:262‐270^30^	To address neuropsychological functioning across different states of BD and to determine relationships among clinical features, neuropsychological performance, and psychosocial functioning	‐ 30 patients with BP‐I or BP‐II and major depression ‐ 34 patients with manic or hypomanic BP‐I or BP‐II ‐ 44 patients with euthymic BP‐I or BP‐II ‐ 30 healthy controls	GAF	Psychosocial functioning	Patients with BP‐I and BP‐II showed decreased performance in verbal memory and executive function compared with healthy subjects, suggesting stability of chronicity of cognitive deficits across bipolar states. Patients with a history of psychotic symptoms, BP‐I, a longer duration of illness, and a large number of manic episodes were more likely to show neuropsychological disturbances. These cognitive difficulties in patients with BD, especially those related to verbal memory, may help explain the impairment in daily functioning, even during remission
Martinez‐Arán 2007 Bipolar Disord 9:103‐113^31^	To identify factors among clinical, neuropsychological, and pharmacologic variables that could be associated with the functional outcome in patients with euthymic BP‐I or BP‐II	‐ 46 high‐functioning patients with BP‐I or BP‐II ‐ 31 low‐functioning patients with BP‐I or BP‐II ‐ 35 healthy controls	GAF	Psychosocial functioning	Cognitive factors may contribute to psychosocial outcome in bipolar disorder. Cognitive impairments were most evident in low‐functioning patients. Differences relative to cognitive dysfunctions between high and low‐functioning patients seem to be independent of illness severity. Verbal memory dysfunction seems to be a good predictor of psychosocial outcome in patients with euthymic BP‐I or BP‐II
Martino 2008 J Affect Disord 109:149‐156^32^	To examine if the extension and severity of cognitive impairments are homogeneously distributed in patients with euthymic BP‐I or BP‐II	‐ 50 patients with euthymic BP‐I or BP‐II ‐ 30 healthy controls matched by age and y of education	GAF	Psychosocial functioning	The extension and severity of cognitive impairments was heterogeneous in patients with BD, explaining the variability in functional outcomes observed in patients with BP‐I or BP‐II
Martino 2011 J Nerv Ment Dis 199:459‐464^33^	To compare neurocognitive functioning between patients with euthymic BP‐I or BP‐II and healthy controls	‐ 48 patients with euthymic BP‐I ‐ 39 patients with euthymic BP‐II ‐ 39 healthy controls matched by age and y of education	GAF	Psychosocial functioning	Patients with euthymic BP‐II showed impairments in psychomotor speed, verbal memory, and executive functions, reproducing closely the profile and magnitude of cognitive deficits of patients with BP‐I. The association between neurocognitive impairments and psychosocial functioning in patients with BP‐II might be as strong as that consistently found in patients with BP‐I
Martino 2014 J Affect Disord 167:118‐124^34^	To expand on previous findings about the prevalence of clinically significant cognitive impairments in a larger sample of patients with BP‐I or BP‐II that meet strict criteria of euthymia	‐ 100 patients with euthymic BP‐I or BP‐II ‐ 40 controls matched by age and y of education	GAF	Psychosocial functioning	Cognitive impairments were heterogeneous in patients with euthymic BP‐I or BP‐II, which could contribute to our understanding of the differences observed in functional outcome
Schoeyen 2013 Bipolar Disord 15:294‐305^35^	To determine the role of premorbid IQ, current IQ, decline in IQ, premorbid function, course of the illness, and demographic characteristics in occupational outcome measured as receipt of disability benefit in BD patients	‐ 144 patients with BP‐I ‐ 70 patients with BP‐II ‐ 12 patients with BP‐NOS	GAF	‐ GAF symptoms ‐ GAF functioning	Low GAF scores indicated that the BD population was both symptomatically and functionally affected. Severe clinical course of BD was associated with receipt of disability benefit. Occupational outcome was unrelated to premorbid adjustment scale, premorbid and current IQ, or decline in IQ, suggesting that the persistence of severe clinical symptoms, rather than global cognitive functioning, determines occupational outcome in patients with BP‐I, BP‐II, or BP‐NOS
FAST					
Aparicio 2017 Acta Psychiatr Scand 135:339‐350^38^	To examine emotion processing in patients with euthymic BP‐I vs healthy controls to determine associations between emotion processing and psychosocial functioning	‐ 60 patients with euthymic BP‐I ‐ 60 healthy controls	FAST	6 FAST domains ‐ Autonomy ‐ Occupational functioning ‐ Cognitive functioning ‐ Financial issues ‐ Interpersonal relationships ‐ Leisure time	Patients with euthymic BP‐I showed emotion processing deficits, especially in the subdomains related to higher‐level social cognitive abilities
Baş 2015 J of Affec Dis 174:336‐341^39^	To evaluate the extent to which cognitive functions, neurological soft signs, and mood symptoms contribute to social disability in patients with euthymic BP‐I	‐ 60 patients with BP‐I ‐ 41 controls matched by age and sex	FAST	6 FAST domains	Residual depressive symptoms and verbal memory impairments were the most prominent factors associated with the level of functioning in patients with euthymic BP‐I
Jiménez 2012 J Affect Disord 136:491‐497^40^	To investigate the functional impact of trait impulsivity in patients with euthymic BP‐I or BP‐II	‐ 138 patients with euthymic BP‐I or BP‐II	FAST	6 FAST domains	Impulsivity, depressive symptoms, and the number of hospitalizations are associated with overall functional impairment in patients with euthymic BP‐I or BP‐II
Kapczinski 2016 Rev Bras Psychiatr 38:201‐206^41^	To study if cognitive and global functioning impairments are associated with the severity of depressive symptoms in patients with BP‐I or BP‐II and depression	‐ 100 patients with BP‐I or BP‐II and depression ‐ 70 healthy controls matched by age and sex	FAST	Functional impairment	Variation in global functioning and cognition, especially in working memory and executive function, was associated with the severity of depressive symptoms observed among patients with depressive BP‐I or BP‐II
Rosa 2010 Value Health 13:984‐988^42^	To assess specific life domains of functioning, as well as the overall functioning across different mood states (hypomania, depression, or euthymia), in patients with BP‐I or BP‐II compared with healthy controls	‐ 68 patients with euthymic BP‐I or BP‐II ‐ 31 patients with hypomanic BP‐I or BP‐II ‐ 32 patients with depressive BP‐I or BP‐II ‐ 61 healthy controls	FAST	6 FAST domains	Patients with BP‐I or BP‐II and depressive or manic episodes experienced poor psychosocial functioning that persisted in an attenuated form during periods of remission. The results highlight the importance of treating both the symptoms of mania and depression aggressively, suggesting that treatment should focus on rehabilitative measures to improve functioning when patients are euthymic
Samalin 2017 J Affect Disord 210:280‐286^43^	To examine a comprehensive model based on structural equation modeling that integrates the interrelationships between residual depressive symptoms, sleep disturbances, and self‐reported cognitive impairment as determinants of psychosocial functioning in real‐life conditions in a sample of patients with euthymic BP‐I and BP‐II	‐ 468 patients with euthymic BP‐I or BP‐II	FAST	6 FAST domains	Residual depressive symptoms and perceived cognitive performance had a direct impact on the functioning of patients with BP‐I or BP‐II during interepisodic times. Sleep disturbances seemed to be indirectly associated with functional impairment
GAF and FAST					
Rosa 2007 Clin Pract Epidemiol Ment Health Jun 7;3:5^22^	To validate the Spanish version of the FAST for its use as an instrument to assess functional impairment in patients with BP‐I or BP‐II	‐ 101 patients with BD ‐ 61 healthy controls	FAST and GAF	GAF Global functioning FAST ‐ 6 FAST domains	The FAST demonstrated strong psychometric properties and had the sensitivity to differentiate among mood states
GAF and scales other than FAST					
Miguélez‐Pan 2014 Psicothema 26:166‐173^36^	To determine the executive functioning profile of a sample of outpatients with euthymic BP‐I, and to explore the complex relationship between differentiated executive processes and multiple dimensions of functional outcome	‐ 34 patients with euthymic BP‐I ‐ 31 healthy controls matched by sex, age, and educational level	GAF Self‐reported SFS	GAF ‐ Overall functioning SFS ‐ Withdrawal ‐ Interpersonal behavior ‐ Prosocial activities ‐ Recreation ‐ Independence‐performance ‐ Independence‐competence ‐ Employment/ occupation	Patients with euthymic BP‐I presented mild deficits in the mental flexibility, verbal and nonverbal fluency, set‐shifting, and planning components of executive functioning, suggesting that the functional complaints often reported by patients with BP‐I might derive from their executive neuropsychological impairment. Besides possible residual affective symptoms, persistent deficits in planning and other action‐directing components of executive ability may account for their frequent functional, occupational, and social difficulties
Other functional scales					
Nilsson 2012 J Behav Ther Exp Psychiat 43:1104‐1108^44^	To examine the relationship between early maladaptive schema and functional impairment	‐ 49 patients with euthymic BD	YSQ‐S3 WSAS	YSQ‐S3 ‐ Disconnection and rejection ‐ Impaired autonomy and performance ‐ Other‐directedness ‐ Overvigilance and inhibition ‐ Impaired limits ‐ WSAS ‐ Work ability ‐ Home management ‐ Social leisure activities ‐ Private leisure activities ‐ Interpersonal relationships	Social isolation, failure to achieve, dependence, vulnerability to harm and illness, emotional inhibition, insufficient self‐control, and pessimism early maladaptive schemas likely play a considerable role in functional impairment
Sole 2012 Acta Psychiatr Scan 125:309‐317^45^	To ascertain whether patients with strictly defined euthymic BP‐II would present neurocognitive disturbances, and to evaluate the impact of the disturbances on functional outcome	‐ 43 patients with euthymic BP‐II ‐ 42 healthy controls	SOFAS	‐ Social functioning ‐ Occupational functioning	Patients with euthymic BP‐II presented cognitive impairments that may affect psychosocial functioning. Patients with BP‐II performed worse than healthy controls in attention, executive functions, and on most measures of verbal learning and memory. Trail‐making executive function abilities and subthreshold depressive symptomatology predicted the functional outcome of these patients
Wingo 2010 Bipolar Disord 12:319‐326^46^	To determine if the functional recovery of patients with euthymic or residually depressed BP‐I or BP‐II is associated with superior neurocognitive functioning, younger age, or more educational, professional, and social accomplishment, including being married	‐ 65 patients with euthymic or residually depressed BP‐I or BP‐II	RSI VSI	‐ RSI ‐ Independent living ‐ Semi‐independent living ‐ Dependent living ‐ VSI ‐ Occupational functioning ‐ Full‐time employed ‐ Part‐time employed ‐ Volunteer ‐ Leave of absence ‐ Unemployed ‐ Disabled	More years of education, being married, and fewer years from illness onset were significantly and independently associated with functional recovery, even after adjusting for current depressive symptoms, BD subtype, and the presence of psychiatric comorbidities. Depression‐prone patients with BP‐II, those with even mild residual depressive symptoms, and those taking antidepressants were less likely to achieve functional recovery

BD, bipolar disorder; BP‐I, bipolar I disorder; BP‐II, bipolar II disorder; BP‐NOS, bipolar disorder not otherwise specified; FAST, Functioning Assessment Short Test; GAF, Global Assessment of Functioning; RSI, Residential Status Index; SFS, Social Functioning Scale; SOFAS, Social and Occupational Functioning Assessment Scale; VSI, Vocational Status Index; WSAS, Work and Social Adjustment Scale; YSQ‐S3, Young Schema Questionnaire Short Form Version 3.

**Table 2:** Longitudinal studies summary

Study	Objective	Patients and interventions	Functional scale and timing of assessment	Functional domains	Conclusions
Observational—FAST					
Bonnín 2014 J Affec Dis 160:50‐54^49^	To explore if verbal memory mediates the relationship between subthreshold depressive symptoms and functional outcome at baseline in euthymic patients with BP‐I or BP‐II followed for 1 y	‐ 111 euthymic patients with BP‐I and BP‐II	FAST ‐ Baseline ‐ 6 mo ‐ 1 y	6 FAST domains	A multivariate model confirmed the role of verbal memory as a mediator in the relationship of subthreshold depressive symptoms and functional outcome, suggesting that neurocognition plays a key role in the prediction of functional outcome
Mora 2016 Compr Psychiatry 71:25‐32^50^	To investigate the progression of cognitive performance and psychosocial functioning in lithium responders over a 6‐y period for a population of euthymic BP‐I and BP‐II that were on lithium therapy at the time of enrollment	At baseline ‐ 44 euthymic patients with BP‐I and BP‐II ‐ 46 healthy matched controls ‐ After 6 y ‐ 8 patients with BP‐I on lithium monotherapy ‐ 2 patients with BP‐II on lithium monotherapy ‐ 10 controls matched by age, sex, and y of education	FAST ‐ Baseline ‐ 6 y	6 FAST domains	Executive functioning, attention, processing speed, and verbal memory cognitive domains were impaired in patients with BP‐I and BP‐II who were excellent lithium responders. Some cognitive domains did not significantly change over time, suggesting that the deficits took place in the early stages of the illness and did not worsen during long‐lasting lithium treatment. Poor cognitive performance was associated to chronicity and poor psychosocial and occupational adjustment
Rosa 2011 Bipolar Disord 13:679‐686^51^	To assess 6‐month functional outcome and the changes that can occur at 4 time periods in a sample of Spanish patients with BP‐I or BP‐II after an acute episode or subsyndromal state	‐ 97 patients with BP‐I or BP‐II	FAST ‐ Baseline ‐ 21 days ‐ 3 mo ‐ 6 mo	6 FAST domains	Although many patients experienced syndromal remission, only a minority reached normal levels of functioning in multiple areas, even after receiving specialized mental healthcare
Rosa 2012 Acta Psychiatr Scand 125:335‐341^52^	To evaluate functional outcome in first‐episode vs multiple‐episode patients with BP‐I, BP‐II, or BP‐NOS in a 12‐month follow‐up study	‐ 60 first mood episode BP‐I, BP‐II or BP‐NOS patients ‐ 59 multiple‐episode patients with BP‐I, BP‐II, or BP‐NOS	FAST ‐ Baseline ‐ 6 mo ‐ 1 y	6 FAST domains	Patients with first episode experienced greater functioning in multiple domains than those with multiple episodes
Strejilevich 2013 Acta Psychiatr Scand 128:194‐202^53^	To identify psychopathological factors associated with long‐term functional outcome in euthymic patients with BP‐I or BP‐II followed for up to 3 y, and to test new mood instability and symptom intensity measures of functional recovery	‐ 55 euthymic patients with BP‐I or BP‐II	FAST ‐ Baseline ‐ Mean follow‐up of 3 y	6 FAST domains	A significant number of patients with BP‐I and BP‐II did not return to their former functioning level, even after receiving the best standard of care and achieving clinical remission. New methodologies, including subsyndromal symptoms and mood instability parameters, should be used to test for new treatments for functional recovery that may draw correlations between cognitive functions and BD models
Observational—FAST and GAF					
Bonnín 2010 J Affect Dis 121:156‐160^54^	To assess which clinical and neurocognitive variables would best predict the functional outcome of euthymic patients with BP‐I or BP‐II in a 4‐y follow‐up study	‐ 32 euthymic patients with BP‐I or BP‐II	GAF at baseline and endpoint FAST at 4‐y endpoint	GAF ‐ Overall functional outcome FAST ‐ Occupational functioning ‐ Interpersonal function ‐ Cognitive functioning	Subdepressive symptomatology and neurocognitive performance at baseline correlated with long‐term psychosocial functioning. Verbal memory plays a role in overall functioning and working memory influences long‐term occupational outcome
Mora 2013 Psychol Med 43:1187‐1196^55^	To assess if cognitive deficits remain stable regardless of the clinical course of the illness, and determine if this impairment could be related to the psychosocial adaptation at the end of the 6‐y follow‐up period for a population of euthymic patients with BP‐I or BP‐II that were on lithium therapy at the time of enrollment	‐ 19 euthymic patients with BP‐I on lithium therapy at the time of enrollment ‐ 9 euthymic patients with BP‐II on lithium therapy at the time of enrollment ‐ 26 healthy controls matched by sex, age, and y of education	GAF at baseline and endpoint FAST at 6‐y endpoint	GAF ‐ Global functioning FAST ‐ 6 FAST domains	Executive functioning, inhibition, processing speed, and verbal memory were impaired in euthymic patients with BP‐I or BP‐II. Although cognitive deficits remained stable throughout follow‐up, they had enduring negative effects on the psychosocial adaptation of patients
Observational—GAF and scales other than FAST					
Martino 2017 J Nerv Ment Dis 205:203‐206^25^	To determine the long‐term functional outcome of euthymic patients with BP‐I or BP‐II followed for ≥ 48 mo under naturalistic conditions of treatment	‐ 55 euthymic patients with BP‐I or BP‐II	GAF Functional recovery self‐assessment Employment status ‐ Baseline ‐ ≥48 mo when they were euthymic	GAF ‐ General Functioning ‐ Functional recovery ‐ Self‐reported measure of functional recovery (Yes/ No) Employment status ‐ Full‐time ‐ Part‐time ‐ Unemployed/ disabled	Patients showed a better level of psychosocial functioning and functional recovery at the end of the follow‐up period than at the study entry. The study provided preliminary evidence that functional outcome tends to be stable over time in the middle course of BD
Tabares‐Seisdedos 2008 J Affect Disord 109:286‐299^56^	To study if neurocognition and clinical factors are significant predictors of functioning in patients with schizophrenia or BP‐I after 1 y, and to determine if the relationships between neurocognition and functional measures are different for these patient populations	‐ 43 patients with BP‐I ‐ 47 patients with schizophrenia ‐ 25 healthy controls	GAF WHODAS ‐ Baseline ‐ 1 y	GAF ‐ Overall functioning WHODAS ‐ Personal care ‐ Occupational functioning ‐ Family functioning ‐ Social functioning	Neuropsychological performance was the principal longitudinal predictor of functioning in both disorders. Baseline neurocognition and cognitive changes over 12 mo predicted changes in functioning over the same period in patients with BP‐I
Tohen 2003 Am J Psychiatry 160:2099‐2107^57^	To follow patients for nearly 4 y to quantify new illness episodes and time to recovery predictors in patients with BP‐I hospitalized with manic or mixed episodes	‐ 166 patients with BD hospitalized for their first manic or mixed episode	GAF MLCI MVSI ‐ Assessments at baseline, weekly until discharge, and at 6, 12, 24, 36, and 48 mo after discharge	GAF ‐ Global functioning MLCI ‐ Residential status MVSI ‐ Occupational level functioning	High proportions of patients initially hospitalized with BP‐I encountered substantial levels of morbidity, comorbidity, and dysfunction in the early years of their course; 28% remained symptomatic, only 43% achieved functional recovery, and 57% had new illness episodes. Current treatments for BD, although effective in facilitating early syndromal recovery, provide incomplete long‐term protection against subsyndromal symptoms, switches, relapses, or recurrences and have a particularly limited impact on functional recovery among patients requiring early hospitalization
Observational—other functional scales					
Burdick 2010 Acta Psychiatr Scand 122:499‐506^59^	To identify which cognitive domains bore significant associations with global, social, or work functioning at a 15‐y follow‐up, and determine whether the severity of recent depressive symptoms, the presence of recent mania, course of illness markers, or medication status contributed additional predictive power in explaining functional disability in patients with BP‐I	‐ 33 patients with BP‐I chosen based on hospital records that met research criteria	Levenstein Global Outcome Scale Strauss‐Carpenter Outcome Scale ‐ Assessment done only at 15‐y endpoint	Levenstein Overall functioning ‐ Good ‐ Moderately impaired ‐ Very poor Strauss‐Carpenter Outcome Scale ‐ Work disability ‐ Social adjustment	Recent depressive symptoms, a greater number of hospitalizations, and processing speed deficits correlated with the functional outcome of patients with BP‐I evaluated 15 y after an index manic episode. Processing speed deficits contribute to poor global functioning and social adaptation, while verbal learning and memory impairment influence occupational status, even after controlling for recent affective symptoms, course of illness features, other cognitive measures, and medication load
Gilbert 2010 J Affect Disord 124:324‐328^58^	To use an innovative, sensitive method for assessing cognitive function to predict employment trajectory in patients with BP‐I treated for 15‐43 mo	‐ 154 patients with BP‐I openly treated with mood stabilizers, typical and atypical antipsychotics, antidepressants, benzodiazepines, or stimulants	SCI‐MOODS ‐ Baseline ‐ 15‐43 mo	SCI‐MOODS cognitive and daily functioning ‐ Memory ‐ Decision making ‐ Concentration ‐ Mental fitness	The ability to predict employment trajectory using the cognitive and daily functioning questions from the SCI‐MOODS points to the sensitivity of this measure and may suggest that cognitive problems are more likely to predict employment trajectory if assessed in the context of specific limitations in functioning
Goldberg 2005 J Affect Disord 89:79‐89^60^	To determine if patients with bipolar or psychotic depression would express poorer life satisfaction on a 7‐ to 8‐y follow‐up vs patients originally hospitalized for unipolar depression, and assess if subjective life satisfaction ratings would reflect objective functional outcome more strongly among nonpsychotic unipolar depression patients than in more severe patients	‐ 35 patients with bipolar mania ‐ 95 patients with unipolar nonpsychotic depression ‐ 27 patients with unipolar psychotic depression	Levenstein Global Outcome Scale Strauss‐Carpenter Outcome Scale ‐ Baseline ‐ 2 y ‐ 4.5 y ‐ 7‐8 y	Levenstein Global Outcome Scale ‐ Psychosocial adjustment Strauss‐Carpenter Outcome Scale ‐ Index of work functioning expressed as effectiveness in an occupation, as a student or homemaker	While objective life satisfaction closely paralleled objective global, occupational, and social adjustment in nonpsychotic unipolar depression, great disparity was evident between subjective and objective outcomes among bipolar and unipolar psychotic depression patients
Loftus 2006 J Nerv Ment Dis 194:967‐970^61^	To examine the impact of Axis II personality disorders and other clinical factors on functional morbidity in a sample of patients with euthymic BP‐I approximately 1 y after hospital discharge	‐ 4 psychiatric hospital in patients with BP‐I ‐ 47 outpatients with BP‐I	Multidimensional Scale for Independent Functioning ‐ Baseline ‐ 1 y	‐ Work environment ‐ Educational, vocational training environment ‐ Residential environment	Depressive symptoms predicted poorer social/leisure adjustment and the ability of patients with BP‐I to live independently over a short‐term follow‐up period
Van Riel 2008 World J Biol Psychiatry 9:313‐320^62^	To determine if patients seeking treatment for manic or mixed bipolar episode followed for 1 y would have higher severity of manic and depressive symptoms, mixed mania, comorbid substance misuse, and poorer psychosocial functioning at baseline	‐ 517 patients seeking treatment for manic or mixed bipolar episode identified as nontreatment responders after 12 mo follow‐up ‐ 2856 patients seeking treatment for manic or mixed bipolar episode identified as responders after 12‐mo follow‐up	SLICE of LIFE ‐ Baseline ‐ Up to 12‐mo follow‐up	‐ Work functioning ‐ Life satisfaction	Prospectively defined chronic mania, in terms of poor treatment response and persistence of significant manic symptoms, during prospective follow‐up of up to 12 mo, was associated to several clinical and psychosocial predictors, including lower severity of manic symptoms at baseline, presence of delusions or hallucinations, shorter duration of the current episode, and impairment in social and occupational activity. Substance misuse and choice of drugs did not predict chronicity. Rather than the severity or the duration of manic symptoms, the presence of psychotic symptoms, and social and occupational functioning are the most important predictors of chronicity in mania
Interventional—FAST					
Torrent 2013 Am J Psychiatry 170:852‐859^69^	To assess the efficacy of functional remediation, a novel intervention program, on functional improvement in a sample of euthymic patients with BP‐I or BP‐II followed for 6 mo	239 euthymic BP‐I or BP‐II patients randomly assigned 1:1:1 to 21 wk of intervention ‐ 77 received functional remediation ‐ 82 received psychoeducation ‐ 80 received treatment as usual	FAST ‐ Baseline ‐ 6 mo	6 FAST domains	The functional remediation program proved to be effective in enhancing functioning in patients with BP‐I or BP‐II; significant improvements were seen in occupational and interpersonal functioning. A combination of medication and functional remediation for patients with relevant disabilities in daily life may ultimately improve the outcome of patients suffering from BD
Interventional—GAF and other functional scales					
de Barros Pellegrinelli 2013 Acta Psychiatr Scand 127:153‐158^67^	To evaluate the efficacy of a psychoeducational approach on symptomatic and functional recovery of euthymic patients with BP‐I or BP‐II followed 12 mo after the end of the treatment, and to identify factors that might influence the efficacy of psychoeducational interventions	55 euthymic patients with BP‐I or BP‐II receiving pharmacologic treatment ‐ 32 received 21 psychoeducation sessions ‐ 23 received 21 relaxation sessions	GAF WHOQOL–BREF SAS‐SR ‐ Baseline ‐ After 8 sessions ‐ After 16 sessions ‐ 6 mo after end of treatment ‐ 12 mo after end of treatment	GAF ‐ Functioning level ‐ WHOQOL–BREF ‐ Environmental domain ‐ Social domain SAS‐SR ‐ Social components	Nonadherence to treatment correlated with worse functioning outcome, social adjustments, sociability, and clinical global impressions. Sixteen psychoeducational sessions seem to be ineffective to prevent mood episodes or improve functioning in a sample of patients with BP‐I and BP‐II
Interventional—other functional scales					
Chengappa 2005 Bipolar Disord 7:68‐76^66^	To determine if patients with BP‐I who recovered from mania would achieve greater improvements in ratings of psychosocial functioning or occupational status after 1 y	Post hoc analysis of double‐blind, placebo‐controlled studies where 139 acutely manic patients with BP‐I received olanzapine 10 mg daily monotherapy for 3 wk followed by a 49‐week open extension ‐ 115 pure mania patients with BP‐I ‐ 24 mixed state mania and depression patients with BP‐I	Self‐reported Medical Outcomes Study SF‐36 psychosocial function and health‐rating questionnaire ‐ Baseline ‐ Weekly during the 3‐week double‐blind phase ‐ At the end of the first, third, and fifth week of the open‐label extension ‐ Monthly up to 1 y	SF‐36 questionnaire ‐ Limitations in physical, social, and major role activities because of poor health	Only 43% of the patients achieved functional recovery, defined as achieving occupational level and residential status that matched or exceeded the preintake level. Symptomatic remission and sustained clinical recovery were achieved only by some manic patients and after mo of delay
Malempati 2015 J Nerv Ment Dis 203:58‐64^68^	To investigate the efficacy and functional recovery associated with aripiprazole adjunct therapy in patients with BP‐I and BP‐II depression over 2 y	‐ 40 patients with BP‐I and BP‐II treated with aripiprazole 5 to 15 mg adjunct to a mood stabilizer	Sheehan Disability Scale ‐ Baseline ‐ 6 mo ‐ 1 y ‐ 2 y	‐ Family life functional recovery ‐ Social life functional recovery ‐ Work/school life	All patients made a complete functional recovery after 2 y as measured by the Sheehan Disability Scale. Aripiprazole adjunct treatment is safe and effective as an acute and maintenance therapy for BD
Weinstock 2010 Compr Psychiatry 51:497‐503^70^	To evaluate family functioning, social support, and functional impairment as predictors of mood symptoms 1 y following acute phase treatment for BP‐I	92 hospitalized or partially hospitalized patients with BP‐I randomly assigned to outpatient treatments for 4 mo during the acute phase of the study, followed by a 24‐month pharmacotherapy acute phase ‐ pharmacotherapy alone ‐ pharmacotherapy plus multifamily psychoeducation group therapy ‐ pharmacotherapy plus individual family therapy Many patients were lost to follow‐up; 34 patients included in the final data set received pharmacotherapy alone, and 6 received pharmacotherapy plus family therapy	UCLA SAS ISEL Family Assessment Device (FAD) ‐ Acute phase treatment completion ‐ 1 y	UCLA SAS ‐ Functional impairment (involvement of activities outside the home) ‐ ISEL ‐ Social support FAD ‐ Family functioning‐	After controlling for the effects of family functioning and functional impairment, social support emerged as a unique predictor of depression severity 1 y following acute phase treatment for BP‐I. The combination of low social support and residual symptomatology may increase the risk of depression in bipolar illness. These data suggest that maintenance therapies focused on improving the level of social support might be especially important to consider in the management of bipolar depression

BD, bipolar disorder; BP‐I, bipolar I disorder; BP‐II, bipolar II disorder; BP‐NOS, bipolar disorder not otherwise specified; FAST, Functioning Assessment Short Test; GAF, Global Assessment of Functioning; ISEL, Interpersonal Support Evaluation List; MLCI, Modified Location Code Index; MVSI, Modified Vocational Status Index; SAS, Social Attainment Survey; SAS‐SR, Social Adjustment Scale Self‐Report; SCI‐MOODS, Structured Clinical Interview for Mood Spectrum; SLICE of LIFE, Streamlined Longitudinal Interview Clinical Evaluation From the Longitudinal Interval Follow‐up Evaluation; WHODAS, World Health Organization Disability Assessment Schedule; WHOQOL‐BREF, Shortened version of the Quality of Life Scale of the World Health Organization Quality of Life Assessment.

### 3.3 Overview of selected studies

The eligible articles included 20 cross‐sectional studies (50%, 20/40; Table 1) and 20 longitudinal studies (50%, 20/40; Table 2). Most of the studies (78%, 31/40) were published after 2007. Twenty studies were conducted in Spain, eight in the United States, five in Argentina, two in Brazil, and one each in Denmark, France, Greece, Norway, and Turkey.

Across studies, a total of 24 different functional outcome assessments were identified, including 13 clinician‐rated scales, 7 self‐reported scales, and 4 indices based on residential and vocational data (Table 3). The most commonly used scales were the Global Assessment of Functioning^21^ (GAF), which was used in 17 studies (43%), and the Functioning Assessment Short Test^22^ (FAST), which was used in 15 studies (38%), both of which are clinician‐rated scales. Although the GAF, developed in 1976, dominated the early literature, the FAST, introduced in 2007, was used in nearly as many studies as the GAF in the last 10 years. Two clinician‐rated scales, the Levenstein Global Outcome Scale^23^ (LGOS) and the Strauss‐Carpenter Outcome Scale^24^ (SCOS) were used in two studies each. The remaining nine clinician‐rated scales were each used in only one study. All seven self‐reported functioning scales were used in only one study each. One study (3%) utilized both the Residential Status Index (RSI) and Vocational Status Index (VSI), while another utilized the Modified Location Code Index (MLCI) and Modified Vocational Status Index (MVSI).

**Table 3:** Functional scales used in the 40 included studies

Scale	Publication Year	Description	Studies, n (%)
Clinician‐rated			
Global Assessment of Functioning^21^ (GAF)^a^	1976	The GAF is a single rating scale for evaluating overall functioning of a subject over a specified time period on a scale ranging from 1 for the hypothetically sickest individual to 100 for the hypothetically healthiest individual	17 (43)
Functioning Assessment Short Test^22^ (FAST)	2007	The FAST evaluates functional impairment in patients with mental disorders based on the following 6 domains: autonomy, occupational functioning, cognitive functioning, financial issues, interpersonal relationships, and leisure time	15 (38)
Levenstein Global Outcome Scale^23^^,^ a	1966	The Levenstein Global Outcome Scale provides a comprehensive measure of psychosocial adjustment for the y preceding each follow‐up based on signs of psychiatric illness, rehospitalization, level of self‐support, role performance, and social relationships	2 (5)
Strauss‐Carpenter Outcome Scale^24^	1972	The Strauss‐Carpenter Scale rates the psychiatric patients in a continuum of outcome dysfunction related to work, social relations, symptoms, and duration of nonhospitalization	2 (5)
World Health Organization Disability Assessment Schedule^63^ (WHODAS)	1988	The WHODAS assesses the social functioning of patients with a mental disorder based on overall behavior, social role performance, hospitalization status, modifying factors, and global evaluations	1 (3)
Social and Occupational Functioning Assessment Scale^83^ (SOFAS)	1992	The SOFAS focuses exclusively on the patient's level of social and occupational functioning without accounting for the severity of the individual's clinical symptoms	1 (3)
Multidimensional Scale for Independent Functioning^64^ (MSIF)	2003	The MSIF captures the patient's inherent responsibilities, the level of assistance provided by others to maintain the role, and the level of performance in which the patient can successfully fulfill roles in work, educational/vocational training, and residential environments	1 (3)
Streamlined Longitudinal Interview Clinical Evaluation From the Longitudinal Interval Follow‐up Evaluation^65^ (SLICE of LIFE)	1987	The LIFE is an integrated system for assessing the longitudinal course of psychiatric disorders that collects detailed information for six areas to link psychosocial and treatment information to psychiatric status ratings. The areas covered are psychosocial (work, household, student, interpersonal, sexual, satisfaction, recreation, global), psychopathologic, nonpsychiatric medical illness, treatment, overall severity, and narrative account	1 (3)
Shortened version of the Quality of Life Scale of the World Health Organization Quality of Life Assessment^71^ (WHOQOL– BREF)	1998	The WHOQOL‐BREF evaluates treatment efficacy by assessing overall quality of life in the areas of physical health (pain and discomfort, sleep and rest, energy and fatigue, mobility, activities of daily living, dependence on medicinal substances and medical aids, work capacity), psychological health, social relationships (personal relationships, social support, sexual activity), and environment (freedom, physical safety and security, home environment, financial resources, health and social care, opportunities for acquiring new information and skills, participation in and opportunities for recreation/leisure activities, physical environment, and transport)	1 (3)
Young Schema Questionnaire Short Version^48^ (YSQ‐S3)	2005	The YSQ‐S3 uses a Likert‐type ranking to assess 18 schemas, patterns that when triggered make the person feel intense emotions in the areas of disconnection and rejection, autonomy and performance, self‐control, directedness, and overvigilance and inhibition	1 (3)
UCLA Social Attainment Survey^73^	1973	The UCLA Social Attainment Survey rates the social adjustments of patients based on same‐sex peer relationships, leadership in same‐sex peer relations, opposite‐sex peer relations, dating history, sexual experience, outside activities, and participation in organizations	1 (3)
Family Assessment Device^84^ (FAD)	1983	The FAD includes seven scales assessing problem solving, communication roles, affective responsiveness, affective involvement, behavior control, and general functioning	1 (3)
Interpersonal Support Evaluation List^85^ (ISEL)	1983	The ISEL includes the following four subscales: tangible assistance (perceived availability of material aid), appraisal (perceived availability of someone to talk about one's problems), self‐esteem (perceived ability of a positive comparison when comparing one's self to others), and belonging (perceived availability of people with whom one can do things)	1 (3)
Self‐reported			
Self‐reported Social Functioning Scale^37^ (SFS)	1990	The SFS assesses the areas of functioning that are crucial to the community maintenance of individuals with schizophrenia. It provides a detailed assessment of strengths and weaknesses of individuals in comparison with reference groups based on the following seven functional domains: social engagement/withdrawal, interpersonal behavior, prosocial activities, recreation, independence‐competence, independence‐performance, and employment/occupation	1 (3)
Work and Social Adjustment Scale^47^ (WSAS)	2002	The WSAS is a short self‐report questionnaire that measures work and social adjustment on the following five domains: work ability, home management, social leisure activities, private leisure activities, and the ability to form and maintain close relationships	1 (3)
Functional Recovery Self‐assessment^25^^,^ a	2017	The self‐assessment of functional recovery was conceptualized as a binary variable based on the patient response to the question, “Have you reached the level of family, social and work functioning that you had before the onset of your illness?”	1 (3)
Self‐reported Medical Outcomes Study SF‐36 psychosocial function and health‐rating questionnaire^74^	1992	The SF‐36 assesses limitations in physical activities, social activities, bodily pain, general mental health, vitality, general health perception, and usual role limitations due to physical or emotional problems	1 (3)
Structured Clinical Interview for Mood Spectrum (SCI‐MOODS)^26^	1999	The SCI‐MOODS uses a dimensional approach to assess threshold level symptoms of unipolar and bipolar disorder and atypical, temperamental and behavioral characteristics over the lifetime and during the past month. It includes the following four domains: mood, energy, cognition, and rhythmicity and vegetative function	1 (3)
Sheehan Disability Scale^75^	2009	The Sheehan Disability Scale evaluates patient impairment levels in the areas of work/school activities, family relationships, and social functioning	1 (3)
Social Adjustment Scale Self‐Report^72^ (SAS‐SR)	1976	The SAS‐SR surveys the patient's role as a spouse, a parent, and a member of a family unit to assess the patient's performance, interpersonal relationships, frictions, feelings and satisfaction in work, and in social and leisure activities with the extended family	1 (3)
Residential and vocational data			
Residential Status Index (RSI) Vocational Status Index (VSI)^57^	2003	The RSI and the VSI serve as proxies of functional recovery by operationalizing ratings for current residential and vocational status that equate or exceed the patient's previous highest residential and vocational status	1 (3)
Modified Vocational Status Index (MVSI) Modified Location Code Index (MLCI)^86^	1988	The MVSI assesses the level of vocational functioning in a range from full‐time, competitive employment at the expected level to total vocational disability; the MLCI records nine living situations ranging from head of household to hospitalization	1 (3)

a Global scales.

Table 3 lists the 24 scales and functional domains addressed by each. Three scales, the GAF, the LGOS, and the Functional Recovery Self‐assessment^25^ (a 1‐question [yes/no] self‐assessment) only provide a global assessment of general functioning, whereas the remaining 21 scales provide assessments related to specific domains such as work/education, residential life, social relationships, family relationships, psychological health, autonomy and performance, and recreation. Thirteen instruments include an occupational functioning domain. Social life was the second most common functional domain included in the scales. Two scales, the FAST and the Structured Clinical Interview for Mood Spectrum^26^ (SCI‐MOODS), include a cognitive function domain. Most studies used ≥1 domain‐specific scale.

### 3.4 Cross‐sectional studies

Of the 20 cross‐sectional studies included in this review, 9 (all published between 2002 and 2016) used the GAF as the sole functioning scale.^27^, ^28^ Four of these studies compared overall functioning between patients with euthymic BP‐I or BP‐II vs healthy controls,^27^, ^32^, ^33^ and four compared functioning between BP‐I, BP‐II, and/or healthy controls or between patients with BP‐I and BP‐II with different mood states (ie, depression, major depression, mania, hypomania, and euthymia) or functioning (ie, low vs high).^29^, ^30^, ^31^ The remaining study^28^ used the GAF to compare functioning in patients with euthymic BD and patients with schizophrenia. Eight^27^, ^28^ of these nine studies that used only the GAF to measure psychosocial functioning evaluated the association between psychosocial functioning and ≥1 cognitive domain (eg, attention, learning and memory, executive function, language). Five of these studies also assessed correlations between the GAF and demographic^27^, ^31^ and/or clinical variables,^27^, ^28^ such as duration of illness, number of hospitalizations, number and type of episodes, symptom type and severity, and medications. One study also evaluated the relationship between psychosocial functioning and theory of mind, the ability to perceive other people's mental states.^27^ The ninth study^35^ used the GAF to assess functioning and symptoms in patients with BD.

In addition to these nine studies using only the GAF, one study^36^ used the GAF along with the Self‐reported Social Functioning Scale (SFS)^37^ to explore the relationships between executive functioning and both global functioning (GAF) and domain‐specific functioning (SFS; ie, withdrawal, interpersonal behavior, prosocial activities, recreation, independence‐performance, independence‐competence, and employment/occupation) in patients with euthymic BP‐I.

Six cross‐sectional studies^38^, ^39^ used the FAST to assess functioning; all were published between 2010 and 2017. Five of these studies assessed domain‐specific functioning (ie, autonomy, occupational functioning, cognitive functioning, financial issues, interpersonal relationships, and leisure time) in patients with BP‐I vs healthy controls,^38^, ^39^ in patients with BP‐I and BP‐II in remission,^40^, ^43^ or across different mood states in BP‐I and BP‐II.^42^ These studies used the FAST to determine correlations between psychosocial functioning and clinical variables (eg, symptoms, number of hospitalizations, number of episodes, duration of illness, trait‐impulsivity, neurologic soft signs),^38^, ^39^, ^40^ emotion processing,^38^ cognitive functioning (eg, verbal learning and memory, visual memory, executive function, perceived cognitive performance),^38^, ^39^, ^43^ social cognition,^38^ sleep disturbances,^43^ or demographic variables^40^ or to assess overall and domain‐specific functioning in different mood states.^42^ The sixth study that used only the FAST to assess psychosocial functioning^41^ compared overall functioning in healthy controls and patients with BP‐I or BP‐II and assessed the relationship to cognitive functioning and depression severity. In addition to the six studies using only the FAST, one study^22^ used the GAF to assess the validity of the Spanish‐language version of the FAST as a new functional scale in patients with BD.

The remaining three cross‐sectional studies^44^, ^45^ used functional scales other than the GAF and the FAST. One study^46^ used the RSI and VSI to assess the relationship between functional recovery and demographic, neurocognitive, and clinical factors in patients with BP‐I or BP‐II who were euthymic or had residual depression. Another study^45^ used the Social and Occupational Functioning Assessment Scale to assess the relationship between social life and occupational functioning and clinical, sociodemographic, and neurocognitive variables (ie, executive function, attention, verbal fluency, and verbal learning and memory) in patients with strictly defined euthymic BP‐II vs healthy controls. The third study^44^ used the Work and Social Adjustment Scale,^47^ which measures occupational, family, social life, and leisure domains, and the Young Schema Questionnaire Short Version^48^ to examine the relationships between domain‐specific functional impairment and early maladaptive schemas.

### 3.5 Longitudinal observational studies

Fifteen longitudinal observational studies met the inclusion criteria. Five studies (all published between 2011 and 2016) only used the FAST scale^49^, ^50^; two (published between 2010 and 2013) used both the GAF and the FAST^54^, ^55^; three (published between 2003 and 2017) used the GAF along with functional scales other than FAST^25^, ^56^, ^57^; and five (published between 2005 and 2010) used scales other than the GAF and the FAST to assess functioning.^58^, ^59^

Five studies assessed functioning using only the FAST scale. Two of the studies^49^, ^53^ assessed functioning in patients with euthymic BP‐I or BP‐II with a follow‐up of 1‐3 years. The first^53^ used the FAST to evaluate the relationships between overall psychosocial functioning and mood, symptoms, and antipsychotic exposure; the second^49^ used it to assess the relationships between psychosocial functioning and verbal memory and subthreshold depressive symptoms. One study^50^ compared 6‐year functional outcome in patients with euthymic BP‐I or BP‐II on lithium monotherapy vs healthy controls, assessing the relationship between changes in psychosocial functioning and cognitive performance (ie, executive function, inhibition, processing speed, verbal memory) over time in patients who responded to lithium. The two remaining studies, both published by the same research group, evaluated functional outcome in patients with BP‐I and BP‐II 6 months after an acute episode or subsyndromal state^51^ and in patients with BP‐I, BP‐II, and BD not otherwise specified after first or multiple affective episodes.^52^

The two observational studies that used both the GAF and the FAST^54^, ^55^ involved authors from the same institution and program. The studies included baseline assessment of psychosocial functioning using only the GAF, with addition of the FAST at endpoint to derive information related to specific functional domains. The first study^54^ sought to identify clinical and neurocognitive predictors of functional outcome in patients with euthymic BP‐I or BP‐II followed for 4 years. The second study^55^ assessed correlations between cognitive changes, including executive functioning, inhibition, attention, processing speed, verbal memory, and visual memory, and functioning in patients with euthymic BP‐I or BP‐II on lithium therapy at the time of enrollment vs healthy controls followed for 6 years.

One study^57^ used the GAF for severity assessment at baseline and the MLCI and the MVSI to evaluate functional recovery at baseline and follow‐up over 4 years in patients with BP‐I hospitalized for a first manic or mixed episode. A second study^56^ used the GAF along with the WHO Psychiatric Disability Assessment Schedule (WHODAS)^63^ to assess the relationship between global functioning (GAF); personal care, occupational, family, and social functioning (WHODAS); and neurocognition in patients with BP‐I, patients with schizophrenia, and healthy controls over 1 year. A third study^25^ assessed the relationship between functioning and number of previous episodes and the long‐term (4 years) functional outcome of patients with euthymic BP‐I or BP‐II under naturalistic treatment conditions, using the GAF to assess global functioning and a self‐reported assessment to measure functional recovery.

Five studies (published between 2005 and 2010)^58^, ^59^ used functional scales other than the GAF and the FAST to assess functioning in patients with various psychiatric disorders. One of these studies^59^ assessed the relationships between overall functioning using the LGOS, work disability/social adjustment using the SCOS, neurocognitive performance, and depressive symptoms in patients with BP‐I 15 years after an index manic episode. Similarly, the LGOS and SCOS were used in a second study^60^ to evaluate global functioning and work performance/social adjustment, respectively, in patients with bipolar mania, unipolar nonpsychotic depression, and unipolar psychotic depression followed for up to 8 years after an index episode, with the objective of examining longitudinal associations with life satisfaction. A third study^61^ used the Multidimensional Scale for Independent Functioning^64^ to compare social adjustment and independent living in inpatients and outpatients with BP‐I 1 year after hospital discharge and relationships with comorbid personality disorders and other clinical factors. The fourth study used the Streamlined Longitudinal Interview Clinical Evaluation from the Longitudinal Interval Follow‐up Evaluation^65^ to compare work functioning and life satisfaction in patients with manic or mixed bipolar episodes identified as responders or nonresponders 1 year after initiating treatment and determine predictors of treatment nonresponse.^62^ A fifth study^58^ used four questions from the SCI‐MOODS to measure self‐reported cognitive problems (ie, memory, decision‐making, concentration, and mental fitness) and the relationship to employment trajectory in patients with BP‐I treated with mood stabilizers, typical and atypical antipsychotics, antidepressants, benzodiazepines, or stimulants and followed for up to 43 months.

#### 3.5.1 Longitudinal interventional studies

Five longitudinal interventional studies^66^, ^67^ (published between 2005 and 2015) met the inclusion criteria for this review. One study^69^ used the FAST to compare functional improvement in patients with euthymic BP‐I or BP‐II randomized to 21 weeks of a functional remediation program, psychoeducation, or treatment as usual. This study^69^ assessed the efficacy of the functional remediation program with respect to overall psychosocial functioning. A second study^67^ used the GAF, the Shortened version of the Quality of Life Scale of the WHO Quality of Life Assessment,^71^ and the Social Adjustment Scale Self‐Report^72^ to assess the effectiveness of psychoeducation compared with relaxation sessions in improving overall functioning, adjustment to the external environment, and social functioning, respectively, at 6‐ and 12‐month follow‐ups in patients with euthymic BP‐I or BP‐II receiving pharmacologic treatment.

The remaining three longitudinal interventional studies used functional scales other than the GAF or FAST. One study^70^ used the UCLA Social Attainment Survey,^73^ the Interpersonal Support Evaluation List, and the Family Assessment Device to measure functional impairment related to activities outside the home, social support, and family functioning, respectively, as a predictor of mood symptoms in patients with BP‐I 1 year after acute phase treatment consisting of pharmacotherapy alone or in conjunction with multifamily psychoeducation group therapy or individual family therapy. The second study^66^ used the Self‐reported Medical Outcomes Study SF‐36 Psychosocial Function and Health‐rating Questionnaire^74^ to assess improvements in physical, social, and major role activities due to poor health and the association with symptomatic remission and clinical recovery in acutely manic patients with BP‐I receiving daily olanzapine monotherapy in the 49‐week open‐label extension of a 3‐week randomized controlled trial. The third and most recent study^68^ used the Sheehan Disability Scale^75^ to assess functional recovery in family, social and work/school domains over 2 years in patients with BP‐I or BP‐II openly treated with aripiprazole and an adjunct mood stabilizer.

## 4 DISCUSSION

The objectives of this systematic literature review were to identify studies that assessed functioning in patients with BD and describe the functional scales used and their implementation. The results show an increasing number of such studies over the past several decades, suggestive of a growing appreciation for the importance of functional outcomes in this patient population. The 40 cross‐sectional and longitudinal studies identified utilized a total of 24 different functional scales, including global and domain‐specific instruments. However, one global assessment, the GAF, and one domain‐specific assessment, the FAST, have prevailed in the literature.

This is the first systematic literature review to examine the scales used in the assessment of functional outcomes in patients with BD. The focus of earlier systematic literature reviews has been on the relationship between cognitive impairment and functional outcomes in this patient population. A systematic literature review of 21 studies concluded that cognitive function is prognostic of general functioning in patients with BD and may be a better predictor than clinical severity measures.^6^ Similarly, a meta‐analysis of 22 studies reported a statistically significant correlation between neurocognitive and everyday functioning in patients with BD.^19^ Another systematic literature review of 12 studies observed an association between impaired cognition and impaired functioning in patients with BD but noted the need for additional studies using objective, performance‐based, functional measures.^46^ Nearly half of the studies included in the current systematic literature review sought to evaluate the relationship between psychosocial functioning and ≥1 domain of cognitive functioning. A similar number of studies examined the relationship between psychosocial functioning and clinical variables such as symptoms, duration of illness, number of episodes, and number of hospitalizations. A smaller number of studies assessed long‐term global or domain‐specific functional outcomes. Taken together with previous reports, the current systematic literature review confirms a growing interest in evaluating functional outcomes in patients with BD and an increasing demand for objective and domain‐specific scales that can address specific areas of interest such as cognition.^39^

The results of this systematic literature review show that the GAF was the most frequently used scale in cross‐sectional studies and the second most frequently used scale in longitudinal studies. After its introduction for use in the Diagnostic and Statistical Manual of Mental Disorders (DSM), Third Edition in 1980,^76^ the GAF gained widespread use and is now the most widely used clinician rating scale of disability.^77^ Although this literature review did not account for studies before 2000 and after the introduction of the GAF, there are several early studies that utilized the GAF, which likely set precedence for its use in later longitudinal and cross‐sectional studies. One key study used the GAF along with the MVSI and MLCI to measure global functioning, occupational status, and residential status, respectively, over 4 years^78^ in patients with BD; the aim of the study was to identify factors contributing to long‐term outcomes.

The reliability and validity of the GAF, however, depend on the rater's training and expertise, and GAF scores have been observed to correlate more with symptom severity than functional impairment.^79^ The FAST was introduced in 2007 as a simple interview to assess the following 6 domains of functioning considered to be the main problem areas for patients with mental illness, including BD: autonomy, occupational functioning, cognitive functioning, finances, interpersonal relationships, and leisure time.^22^ Results of this literature review show rapid and widespread adoption of the FAST in clinical studies, with use in 7 of 17 cross‐sectional studies, 7 of 12 longitudinal observational studies, and 1 of 4 longitudinal interventional studies published after 2007. The trend toward evaluating multiple domain‐specific aspects of functioning in patients with BD is expected to continue after the official deletion of the GAF from the DSM, Fifth Edition^80^ and the endorsement of the WHO Disability Assessment Schedule Version 2.0 (WHODAS 2.0),^81^ which measures cognition, mobility, self‐care, getting along, life activities, and participation, for clinical evaluation of disability.^82^ We see the adoption of the WHODAS as a valuable addition to the field as it provides more specific measures of functioning. On the other hand, the GAF provides an overall rating that includes functioning which in some cases may be useful because of its ease of use. As with any scale, investigators will need to decide which scale best meets their needs.

The remaining 22 scales of functioning identified in this systematic literature review were used in few studies. It is worth noting that all but 2 of these 22 scales are domain‐specific measures of functioning. The use of many different domain‐specific assessments during the early time frame of this systematic literature review may reflect the lack of availability during that period of a single functional scale capturing all functional domains of interest. Although there was considerable variability across these scales with respect to the nature of included functional domains, work/education, social, and family domains were most widely represented. The prevalence of scales incorporating these domains likely reflects an understanding that work, family, and social/interpersonal engagement are key areas of functional impairment in patients with BD that can persist into clinical remission^5^, ^10^ and affect the well‐being of patients and their families. However, the abundance of unique scales to assess the same functional domains presents a challenge when attempting to synthesize the literature. Although cognitive functioning was not broadly represented in these less widely utilized scales, it is included in the FAST and the WHODAS 2.0.

The results of this systematic literature review further the understanding of the scales used to assess functioning in patients with BD and the nature of the research questions to which they are applied. Such findings are important considering the recognition of different types of functional impairment in this patient population and the persistence of these impairments into symptomatic remission. However, results of this literature review show a relatively small number of interventional studies assessing functional outcomes in patients with BD. Thus, there is a continued need to ensure that functioning of patients with BD is included as an outcome in studies evaluating the effects of treatment interventions.

This systematic review has several limitations. Although the search parameters were designed to be comprehensive, it omitted non‐English articles and articles published before 2000, which could have limited its scope. In several areas of the literature, particularly studies using the GAF and FAST, multiple studies were published by the same research groups or authors at the same institutions. Although an effort was made to omit duplicate publications, it is important to recognize the potential for bias due to multiple publications by the same investigators.

In conclusion, this review shows high utilization of the GAF and FAST scales for assessment of functioning in cross‐sectional observational and interventional studies. Findings indicate interest in evaluating specific functional domains, with emphasis on work/educational, social, family, and cognitive functioning, and determining the relationships between psychosocial functioning, cognitive functioning, and clinical variables. More prospective studies are needed to assess the effect of interventional therapies on functional outcomes in patients with BD.

## DISCLOSURES

Maxine Chen and Jessica J. Madera are employees of Otsuka Pharmaceutical Development & Commercialization, Inc. Heather M. Fitzgerald is an employee of Lundbeck LLC. Mauricio Tohen was an employee of Eli Lilly (1997‐2008) and has received honoraria from or consulted for Abbott, AstraZeneca, Alkermes, Allergan, Bristol Myers Squibb, GlaxoSmithKline, Eli Lilly, Johnson & Johnson, Otsuka, Merck, Sunovion, Forest, Roche, Elan, Lundbeck, Teva, Pamlab, Minerva, Pfizer, Wyeth, and Wiley Publishing; his spouse was a full‐time employee at Eli Lilly (1998‐2013).

## References

[1] Clemente AS , Diniz BS , Nicolato R , et al. Bipolar disorder prevalence: a systematic review and meta‐analysis of the literature. Rev Bras Psiquiatr. 2015;37:155‐161. 25946396 10.1590/1516-4446-2012-1693

[2] Merikangas KR , Jin R , He J‐P , et al. Prevalence and correlates of bipolar spectrum disorder in the world mental health survey initiative. Arch Gen Psychiatry. 2011;68:241‐251. 21383262 10.1001/archgenpsychiatry.2011.12 PMC3486639

[3] Merikangas KR , Tohen M . Epidemiology of bipolar disorder in adults and children In: Tsuang MT, Tohen M, Jones PB, eds. Textbook of Psychiatric Epidemiology. West Sussex, UK: John Wiley & Sons Ltd; 2011:329‐342.

[4] World Health Organization . The Global Burden of Disease: 2004 update. World Health Organization http://www.who.int/healthinfo/global_burden_disease/GBD_report_2004update_full.pdf?ua=1. Accessed June 29, 2018.

[5] Sanchez‐Moreno J , Martinez‐Aran A , Tabarés‐Seisdedos R , Torrent C , Vieta E , Ayuso‐Mateos Jl . Functioning and disability in bipolar disorder: an extensive review. Psychother Psychosom. 2009;78:285‐297. 19602917 10.1159/000228249

[6] Baune BT , Malhi GS . A review on the impact of cognitive dysfunction on social, occupational, and general functional outcomes in bipolar disorder. Bipolar Disord. 2015;17:41‐55. 26688289 10.1111/bdi.12341

[7] Centers for Disease Control and Prevention . The ICF: An Overview. U.S. Department of Health & Human Services https://www.cdc.gov/nchs/data/icd/icfoverview_finalforwho10sept.pdf. Accessed May 15, 2018.

[8] Michalak EE , Yatham LN , Maxwell V , Hale S , Lam RW . The impact of bipolar disorder upon work functioning: a qualitative analysis. Bipolar Disord. 2007;9:126‐143. 17391356 10.1111/j.1399-5618.2007.00436.x

[9] Judd LL , Schettler PJ , Solomon DA , et al. Psychosocial disability and work role function compared across the long‐term course of bipolar I, bipolar II and unipolar major depressive disorders. J Affect Disord. 2008;108:49‐58. 18006071 10.1016/j.jad.2007.06.014

[10] Morselli PL , Elgie R , Cesana BM . GAMIAN‐Europe/BEAM survey II: cross‐national analysis of unemployment, family history, treatment satisfaction and impact of the bipolar disorder on life style. Bipolar Disord. 2004;6:487‐497. 15541064 10.1111/j.1399-5618.2004.00160.x

[11] Bauwens F , Tracy A , Pardoen D , Elst MV , Mendlewicz J . Social adjustment of remitted bipolar and unipolar out‐patients. A comparison with age‐ and sex‐matched controls. Br J Psychiatry. 1991;159:239‐244. 1773240 10.1192/bjp.159.2.239

[12] Shapira B , Zislin J , Gelfin Y , et al. Social adjustment and self‐esteem in remitted patients with unipolar and bipolar affective disorder: a case‐control study. Compr Psychiatry. 1999;40:24‐30. 9924873 10.1016/s0010-440x(99)90072-x

[13] Mitchell PB , Slade T , Andrews G . Twelve‐month prevalence and disability of DSM‐IV bipolar disorder in an Australian general population survey. Psychol Med. 2004;34:777‐785. 15500298 10.1017/s0033291703001636

[14] Thomas SP , Nisha A , Varghese PJ . Disability and quality of life of subjects with bipolar affective disorder in remission. Indian J Psychol Med. 2016;38:336‐340. 27570346 10.4103/0253-7176.185941 PMC4980902

[15] World Health Organization . The world health report 2001: mental health: new understanding, new hope. Geneva, Switzerland: World Health Organization; 2001.

[16] Goossens PJ , Van Wijngaarden B , Knoppert‐van Der Klein EA , Van Achterberg T . Family caregiving in bipolar disorder: caregiver consequences, caregiver coping styles, and caregiver distress. Int J Soc Psychiatry. 2008;54:303‐316. 18720891 10.1177/0020764008090284

[17] Gitlin MJ , Miklowitz DJ . The difficult lives of individuals with bipolar disorder: a review of functional outcomes and their implications for treatment. J Affect Disord. 2017;209:147‐154. 27914248 10.1016/j.jad.2016.11.021 PMC7213058

[18] Wingo AP , Harvey PD , Baldessarini RJ . Neurocognitive impairment in bipolar disorder patients: functional implications. Bipolar Disord. 2009;11:113‐125. 19267694 10.1111/j.1399-5618.2009.00665.x

[19] Depp CA , Mausbach BT , Harmell AL , et al. Meta‐analysis of the association between cognitive abilities and everyday functioning in bipolar disorder. Bipolar Disord. 2012;14:217‐226. 22548895 10.1111/j.1399-5618.2012.01011.x PMC3396289

[20] Liberati A , Altman DG , Tetzlaff J , et al. The PRISMA statement for reporting systematic reviews and meta‐analyses of studies that evaluate healthcare interventions: explanation and elaboration. BMJ. 2009;339:b2700. 19622552 10.1136/bmj.b2700 PMC2714672

[21] Endicott J , Spitzer RL , Fleiss JL , Cohen J . The global assessment scale. A procedure for measuring overall severity of psychiatric disturbance. Arch Gen Psychiatry. 1976;33:766‐771. 938196 10.1001/archpsyc.1976.01770060086012

[22] Rosa AR , Sánchez‐Moreno J , Martínez‐Aran A , et al. Validity and reliability of the Functioning Assessment Short Test (FAST) in bipolar disorder. Clin Pract Epidemiol Ment Health. 2007;3:5. 17555558 10.1186/1745-0179-3-5 PMC1904447

[23] Levenstein S , Klein DF , Pollack M . Follow‐up study of formerly hospitalized voluntary psychiatric patients: the first two years. Am J Psychiatry. 1966;122:1102‐1109. 5909001 10.1176/ajp.122.10.1102

[24] Strauss JS , Carpenter WT . Jr. The prediction of outcome in schizophrenia. I. Characteristics of outcome. Arch Gen Psychiatry. 1972;27:739‐746. 4637891 10.1001/archpsyc.1972.01750300011002

[25] Martino DJ , Igoa A , Scápola M , Marengo E , Samamé C , Strejilevich SA . Functional outcome in the middle course of bipolar disorder: a longitudinal study. J Nerv Ment Dis. 2017;205:203‐206. 28234724 10.1097/NMD.0000000000000583

[26] Fagiolini A , Dell'osso L , Pini S , et al. Validity and reliability of a new instrument for assessing mood symptomatology: the Structured Clinical Interview for Mood Spectrum (SCI‐MOODS). Int J Meth Psych Res. 1999;8:71‐82.

[27] Konstantakopoulos G , Ioannidi N , Typaldou M , Sakkas D , Oulis P . Clinical and cognitive factors affecting psychosocial functioning in remitted patients with bipolar disorder. Psychiatriki. 2016;27:182‐191. 27837572 10.22365/jpsych.2016.273.182

[28] Martínez‐Arán A , Penadés R , Vieta E , et al. Executive function in patients with remitted bipolar disorder and schizophrenia and its relationship with functional outcome. Psychother Psychosom. 2002;71:39‐46. 11740167 10.1159/000049342

[29] Martinez‐Aran A , Vieta E , Colom F , et al. Neuropsychological performance in depressed and euthymic bipolar patients. Neuropsychobiology. 2002;46(Suppl 1):16‐214. 12571428 10.1159/000068016

[30] Martínez‐Arán A , Vieta E , Reinares M , et al. Cognitive function across manic or hypomanic, depressed, and euthymic states in bipolar disorder. Am J Psychiatry. 2004;161:262‐270. 14754775 10.1176/appi.ajp.161.2.262

[31] Martinez‐Aran A , Vieta E , Torrent C , et al. Functional outcome in bipolar disorder: the role of clinical and cognitive factors. Bipolar Disord. 2007;9:103‐113. 17391354 10.1111/j.1399-5618.2007.00327.x

[32] Martino DJ , Strejilevich SA , Scápola M , et al. Heterogeneity in cognitive functioning among patients with bipolar disorder. J Affect Disord. 2008;109:149‐156. 18234352 10.1016/j.jad.2007.12.232

[33] Martino DJ , Igoa A , Marengo E , Scapola M , Strejilevich SA . Neurocognitive impairments and their relationship with psychosocial functioning in euthymic bipolar II disorder. J Nerv Ment Dis. 2011;199:459‐464. 21716059 10.1097/NMD.0b013e3182214190

[34] Martino DJ , Strejilevich SA , Marengo E , Ibañez A , Scápola M , Igoa A . Toward the identification of neurocognitive subtypes in euthymic patients with bipolar disorder. J Affect Disord. 2014;167:118‐124. 24955563 10.1016/j.jad.2014.05.059

[35] Schoeyen HK , Melle I , Sundet K , et al. Occupational outcome in bipolar disorder is not predicted by premorbid functioning and intelligence. Bipolar Disord. 2013;15:294‐305. 23527993 10.1111/bdi.12056

[36] Miguelez‐Pan M , Pousa E , Cobo J , Duno R . Cognitive executive performance influences functional outcome in euthymic type I bipolar disorder outpatients. Psicothema. 2014;26:166‐173. 24755016 10.7334/psicothema2013.111

[37] Birchwood M , Smith J , Cochrane R , Wetton S , Copestake S . The Social Functioning Scale. The development and validation of a new scale of social adjustment for use in family intervention programmes with schizophrenic patients. Br J Psychiatry. 1990;157:853‐859. 2289094 10.1192/bjp.157.6.853

[38] Aparicio A , Santos Jl , Jiménez‐López E , Bagney A , Rodríguez‐Jiménez R , Sánchez‐Morla Em . Emotion processing and psychosocial functioning in euthymic bipolar disorder. Acta Psychiatr Scand. 2017;135:339‐350. 28188631 10.1111/acps.12706

[39] Bas TO , Poyraz CA , Bas A , Poyraz BC , Tosun M . The impact of cognitive impairment, neurological soft signs and subdepressive symptoms on functional outcome in bipolar disorder. J Affect Disord. 2015;174:336‐341. 25545601 10.1016/j.jad.2014.12.026

[40] Jiménez E , Arias B , Castellví P , et al. Impulsivity and functional impairment in bipolar disorder. J Affect Disord. 2012;136:491‐497. 22129768 10.1016/j.jad.2011.10.044

[41] Kapczinski NS , Narvaez JC , Magalhães PV , et al. Cognition and functioning in bipolar depression. Rev Bras Psiquiatr. 2016;38:201‐206. 26870909 10.1590/1516-4446-2014-1558 PMC7194267

[42] Rosa AR , Reinares M , Michalak EE , et al. Functional impairment and disability across mood states in bipolar disorder. Value Health. 2010;13:984‐988. 20667057 10.1111/j.1524-4733.2010.00768.x

[43] Samalin L , Boyer L , Murru A , et al. Residual depressive symptoms, sleep disturbance and perceived cognitive impairment as determinants of functioning in patients with bipolar disorder. J Affect Disord. 2017;210:280‐286. 28068616 10.1016/j.jad.2016.12.054

[44] Nilsson KK . Early maladaptive schemas and functional impairment in remitted bipolar disorder patients. J Behav Ther Exp Psychiatry. 2012;43:1104‐1108. 22743343 10.1016/j.jbtep.2012.05.005

[45] Solé B , Bonnin Cm , Torrent C , et al. Neurocognitive impairment and psychosocial functioning in bipolar II disorder. Acta Psychiatr Scand. 2012;125:309‐317. 21848702 10.1111/j.1600-0447.2011.01759.x

[46] Wingo AP , Baldessarini RJ , Holtzheimer PE , Harvey PD . Factors associated with functional recovery in bipolar disorder patients. Bipolar Disord. 2010;12:319‐326. 20565439 10.1111/j.1399-5618.2010.00808.x PMC3749090

[47] Mundt JC , Marks IM , Shear MK , Greist JH . The work and social adjustment scale: a simple measure of impairment in functioning. Br J Psychiatry. 2002;180:461‐464. 11983645 10.1192/bjp.180.5.461

[48] Young J . Young schema questionnaire—short version (YSQ‐S3). New York, NY: Creative Therapy Centers of New York; 2005.

[49] Bonnín C , González‐Pinto A , Solé B , et al. Verbal memory as a mediator in the relationship between subthreshold depressive symptoms and functional outcome in bipolar disorder. J Affect Disord. 2014;160:50‐54. 24709022 10.1016/j.jad.2014.02.034

[50] Mora E , Portella MJ , Forcada I , Vieta E , Mur M . A preliminary longitudinal study on the cognitive and functional outcome of bipolar excellent lithium responders. Compr Psychiatry. 2016;71:25‐32. 27592139 10.1016/j.comppsych.2016.07.008

[51] Rosa AR , Reinares M , Amann B , et al. Six‐month functional outcome of a bipolar disorder cohort in the context of a specialized‐care program. Bipolar Disord. 2011;13:679‐686. 22085481 10.1111/j.1399-5618.2011.00964.x

[52] Rosa AR , González‐Ortega I , González‐Pinto A , et al. One‐year psychosocial functioning in patients in the early vs. late stage of bipolar disorder. Acta Psychiatr Scand. 2012;125:335‐341. 22283440 10.1111/j.1600-0447.2011.01830.x

[53] Strejilevich SA , Martino DJ , Murru A , et al. Mood instability and functional recovery in bipolar disorders. Acta Psychiatr Scand. 2013;128:194‐202. 23331090 10.1111/acps.12065

[54] Bonnín Cm , Martínez‐Arán A , Torrent C , et al. Clinical and neurocognitive predictors of functional outcome in bipolar euthymic patients: a long‐term, follow‐up study. J Affect Disord. 2010;121:156‐160. 19505727 10.1016/j.jad.2009.05.014

[55] Mora E , Portella MJ , Forcada I , Vieta E , Mur M . Persistence of cognitive impairment and its negative impact on psychosocial functioning in lithium‐treated, euthymic bipolar patients: a 6‐year follow‐up study. Psychol Med. 2013;43:1187‐1196. 22935452 10.1017/S0033291712001948

[56] Tabarés‐Seisdedos R , Balanzá‐Martínez V , Sánchez‐Moreno J , et al. Neurocognitive and clinical predictors of functional outcome in patients with schizophrenia and bipolar I disorder at one‐year follow‐up. J Affect Disord. 2008;109:286‐299. 18289698 10.1016/j.jad.2007.12.234

[57] Tohen M , Zarate CA , Hennen J , et al. The McLean‐Harvard First‐Episode Mania Study: prediction of recovery and first recurrence. Am J Psychiatry. 2003;160:2099‐2107. 14638578 10.1176/appi.ajp.160.12.2099

[58] Gilbert Am , Olino Tm , Houck P , Fagiolini A , Kupfer Dj , Frank E . Self‐reported cognitive problems predict employment trajectory in patients with bipolar I disorder. J Affect Disord. 2010;124:324‐328. 19942294 10.1016/j.jad.2009.11.012 PMC2888870

[59] Burdick KE , Goldberg JF , Harrow M . Neurocognitive dysfunction and psychosocial outcome in patients with bipolar I disorder at 15‐year follow‐up. Acta Psychiatr Scand. 2010;122:499‐506. 20637012 10.1111/j.1600-0447.2010.01590.x PMC2980854

[60] Goldberg JF , Harrow M . Subjective life satisfaction and objective functional outcome in bipolar and unipolar mood disorders: a longitudinal analysis. J Affect Disord. 2005;89:79‐89. 16249035 10.1016/j.jad.2005.08.008

[61] Loftus ST , Jaeger J . Psychosocial outcome in bipolar I patients with a personality disorder. J Nerv Ment Dis. 2006;194:967‐970. 17164638 10.1097/01.nmd.0000243814.35854.10

[62] Van Riel WG , Vieta E , Martinez‐Aran A , et al. Chronic mania revisited: factors associated with treatment non‐response during prospective follow‐up of a large European cohort (EMBLEM). World J Biol Psychiatry. 2008;9:313‐320. 18949649 10.1080/15622970701805491

[63] World Health Organization . WHO PSYCHIATRIC DISABILITY ASSESSMENT SCHEDULE (WHO/DAS). England: Macmillan/Clays; 1988.

[64] Jaeger J , Berns SM , Czobor P . The multidimensional scale of independent functioning: a new instrument for measuring functional disability in psychiatric populations. Schizophr Bull. 2003;29:153‐168. 12908671 10.1093/oxfordjournals.schbul.a006987

[65] Keller MB , Lavori PW , Friedman B , et al. The Longitudinal Interval Follow‐up Evaluation. A comprehensive method for assessing outcome in prospective longitudinal studies. Arch Gen Psychiatry. 1987;44:540‐548. 3579500 10.1001/archpsyc.1987.01800180050009

[66] Roy Chengappa Kn , Hennen J , Baldessarini RJ , et al. Recovery and functional outcomes following olanzapine treatment for bipolar I mania. Bipolar Disord. 2005;7:68‐76. 15654934 10.1111/j.1399-5618.2004.00171.x

[67] de Barros Pellegrinelli K , de O. Costa Lf , Silval K , et al. Efficacy of psychoeducation on symptomatic and functional recovery in bipolar disorder. Acta Psychiatr Scand. 2013;127:153‐158. 22943487 10.1111/acps.12007

[68] Malempati RN . Aripiprazole adjunct treatment in bipolar I or II disorder, depressed state: a 2‐year clinical study. J Nerv Ment Dis. 2015;203:58‐64. 25536100 10.1097/NMD.0000000000000234

[69] Torrent C , Bonnin C , Martínez‐Arán A , et al. Efficacy of functional remediation in bipolar disorder: a multicenter randomized controlled study. Am J Psychiatry. 2013;170:852‐859. 23511717 10.1176/appi.ajp.2012.12070971

[70] Weinstock LM , Miller IW . Psychosocial predictors of mood symptoms 1 year after acute phase treatment of bipolar I disorder. Compr Psychiatry. 2010;51:497‐503. 20728007 10.1016/j.comppsych.2010.02.001 PMC2947345

[71] The WHOQOL Group . Development of the World Health Organization WHOQOL‐BREF quality of life assessment. Psychol Med. 1998;28:551‐558. 9626712 10.1017/s0033291798006667

[72] Weissman MM , Bothwell S . Assessment of social adjustment by patient self‐report. Arch Gen Psychiatry. 1976;33:1111‐1115. 962494 10.1001/archpsyc.1976.01770090101010

[73] Evans JR , Goldstein MJ , Rodnick EH . Premorbid adjustment, paranoid diagnosis, and remission. Acute schizophrenics treated in a community mental health center. Arch Gen Psychiatry. 1973;28:666‐672. 4349538 10.1001/archpsyc.1973.01750350046009

[74] Ware JE Jr . Sherbourne CD. The MOS 36‐item short‐form health survey (SF‐36). I. Conceptual framework and item selection. Med Care. 1992;30:473‐483. 1593914

[75] Arbuckle R , Frye MA , Brecher M , et al. The psychometric validation of the Sheehan Disability Scale (SDS) in patients with bipolar disorder. Psychiatry Res. 2009;165:163‐174. 19042030 10.1016/j.psychres.2007.11.018

[76] American Psychiatric Association . Diagnostic and Statistical Manual of Mental Disorders, 3rd edn Washington DC: American Psychiatric Association; 1980.

[77] Von Korff M , Andrews G , Delves M . Assessing activity limitations and disability among adults In: Regier D, Narrow W, Kuhl E, Kupfer D, eds. The Conceptual Evolution of DSM‐5. Washington DC: American Psychiatric Publishing Inc; 2011:163‐188.

[78] Tohen M , Waternaux CM , Tsuang MT , Hunt AT . Four‐year follow‐up of twenty‐four first‐episode manic patients. J Affect Disord. 1990;19:79‐86. 2142702 10.1016/0165-0327(90)90012-w

[79] Gold LH . DSM‐5 and the assessment of functioning: the World Health Organization Disability Assessment Schedule 2.0 (WHODAS 2.0). J Am Acad Psychiatry Law. 2014;42:173‐181. 24986344

[80] American Psychiatric Nurses Association . Recovery to practice pledge. . http://www.apna.org/i4a/pages/index.cfm?pageID=4606. Accessed February 12, 2016.

[81] American Psychiatric Association . Insurance implications of DSM‐5. Washington, DC: American Psychiatric Association; 2013.

[82] World Health Organization . WHO Disability Assessment Schedule 2.l0 (WHODAS 2.0). Geneva, Switzerland: World Health Organization. Accessed June 1, 2018.

[83] Goldman HH , Skodol AE , Lave TR . Revising axis V for DSM‐IV: a review of measures of social functioning. Am J Psychiatry. 1992;149:1148‐1156. 1386964 10.1176/ajp.149.9.1148

[84] Epstein N , Baldwin L , Bishop D . The McMaster family assessment device. J Marital Fam Ther. 1983;9:171‐180.

[85] Cohen S , Hoberman H . Positive events and social supports as buffers of life change stress. J Appl Soc Psychol. 1983;13:99‐125.

[86] Dion GL , Tohen M , Anthony WA , Waternaux CS . Symptoms and functioning of patients with bipolar disorder six months after hospitalization. Hosp Community Psychiatry. 1988;39:652‐657. 3402925 10.1176/ps.39.6.652

